# Anoctamin-1 in the Juvenile Rat Urinary Bladder

**DOI:** 10.1371/journal.pone.0106190

**Published:** 2014-09-02

**Authors:** Dominika A. Bijos, Marcus J. Drake, Bahareh Vahabi

**Affiliations:** 1 School of Clinical Sciences, University of Bristol, Bristol, United Kingdom; 2 Bristol Urological Institute, North Bristol NHS Trust, Bristol, United Kingdom; 3 Department of Biological, Biomedical and Analytical Sciences, University of the West of England, Bristol, United Kingdom; Monell Chemical Senses Center, United States of America

## Abstract

**Purpose:**

To investigate presence, location and functional role of calcium-activated chloride channel (CaCC) Anoctamin-1 (Ano1) in rat urinary bladder.

**Materials and Methods:**

Bladders from 3 week old Wistar rats were studied. End-point PCR on total mRNA was used to assess the expression of Ano1. Immunofluorescent labelling of whole mount bladder tissue imaged with confocal microscope allowed localization of Ano1 and vimentin immunopositive cells. The effects of CaCC blockers: niflumic acid (NFA) (3,10,30 µM) and 5-Nitro-2-(3-phenylpropylamino)benzoic acid (NPPB) (10, 30 µM) on spontaneous phasic contractile activity of intact (with mucosa) and denuded (without mucosa) detrusor strips were measured under isometric tension in organ baths (n = 141, N = 60).

**Results:**

Ano1 expression was found at mRNA level in mucosa and detrusor layers. Confocal microscopy revealed presence of Ano1 immunopositive cells in mucosa and in detrusor layers; a subpopulation of vimentin positive cells expressed Ano1. Both chloride channel blockers reduced the amplitude and frequency of phasic contractions in denuded and intact strips.

**Conclusions:**

Ano1 is expressed in rat urinary bladder and is present in cells sharing markers with interstitial cells. CaCC blockers reduced phasic activity of the bladder tissue. Ano1 is expressed in the bladder and plays a role in its spontaneous phasic contractile activity.

## Introduction

Urinary bladder exhibits spontaneous phasic contractile activity similar to peristaltic movements of the gastrointestinal tract [1]. The physiological relevance of this phasic activity is not clear, but it may play a role in mediating the tone of the bladder and relaying sensory afferent information [2]. A population of cells similar to interstitial cells of Cajal (ICCs) in the gut have been found in the bladder lamina propria and detrusor layers [3] and it is suggested that these cells play a role in modulating the phasic activity of the detrusor [4]. A heterogeneous population of interstitial cells has been found in the urinary bladder of various species using antibodies to the intermediate filament vimentin, the tyrosine kinase receptor KIT, the platelet-derived growth factor receptor alpha (PDGFRα) and adhesion molecule CD34 [5], [6]. Although these markers are used extensively in bladder research, better cellular markers are needed in order to study the functional role of these cells in the bladder.

Interstitial cells have distinctive functionalities in different regions of the bladder wall [7]. For example, interstitial cells in muscle respond to cholinergic stimulation, whilst interstitial cells in lamina propria are sensitive to ATP and exhibit a calcium-activated chloride current [8]. Despite significant progress in the study of bladder interstitial cells' markers, receptors, ion channels, electrical and calcium signalling, their specific functions in normal bladder function remain elusive [6].

ICCs in the gut act as pacemakers and drive peristalsis, by generating spontaneous transient inward currents which stimulate smooth muscle contractions [9]. Recently, a calcium activated chloride channel (CaCC), Anoctamin-1 (Ano1), was identified specifically expressed on ICCs of the gut in various species [10], [11] and is essential for generation of the slow wave activity of ICCs [10]. The functional role of Ano1 was assessed in the gut using various CaCC blockers such as 5-Nitro-2-(3-phenylpropylamino)benzoic acid (NPPB) and niflumic acid (NFA) [12]. It was demonstrated that the slow wave activity of the ICCs could be inhibited by these mediators [10].

A recent study by Yu et al. (2012) demonstrated that a subpopulation of interstitial cells expressing CD34 also expressed Ano1 in mouse urinary bladder [5]. However, the functional role of these cells was not assessed. The influence of CaCC blockers such as NFA has also been recently assessed on the spontaneous contractions of rat urinary bladder strips [13], but this study did not confirm the expression of Ano1 in the rat bladder.

The aim of this study was to confirm the expression of Ano1 in the rat urinary bladder and assess the role of CaCC blockers in modulating the phasic contraction of intact and denuded bladder strips.

## Materials and Methods

### Tissue Preparation

Urinary bladders were obtained from 21±2 days old Wistar rats killed by procedures in accordance with UK Government regulations (Animals Scientific Procedures Act 1986). This study did not require a UK Home Office project license, because no regulated procedures were carried out. Rats were humanely killed at a designated establishment by cranial concussion followed by cervical dislocation, which is an appropriate method under Schedule 1 of the Act. Bladders were removed and placed in cold Krebs bicarbonate solution (NaCl 118.4 mM, glucose 11.7 mM, NaHCO_3_ 24.9 mM, KCl 4.7 mM, CaCl_2_ 1.9 mM, MgSO_4_ 1.15 mM, KH_2_PO_4_ 1.15 mM). A total of 5 bladders were used for molecular studies and confocal microscopy, and 60 for functional studies.

### Molecular Studies

Total RNA was extracted from mucosa and denuded-detrusor layers of rat bladders using a ReliaPrep RNA Tissue Miniprep system (Promega, USA) according to manufacturer's instructions. Tissues were homogenized using a Bio-Gen PRO200 homogenizer (ProScientific, USA). Following RNA quantification by Nanodrop (ThermoScientific, UK), 1 µg of total RNA was reverse transcribed to single strand cDNA using GoScript Reverse Transcription System (Promega, USA) according to the manufacturer's instructions. cDNA samples were amplified using GAPDH forward (5′ACCCAGAAGACTGTGGATGG 3′) and reverse (5′CACATTGGGGGTAGGAACAC3′) primers (positive control) and specific Ano1 (Accession number NM_001107564.1) forward (5′ TCAATGTCAGCGACTTCCAG 3′) and reverse (5′ TTTGGGGATATCAGGGATCA 3′) primers. Reverse transcription reactions without RNA were included instead of cDNA template as negative controls. All primers were supplied by Invitrogen (UK). PCR amplification consisted of a denaturation step at 95°C for 3 min, 36 cycles of 95°C for 30 sec, 55°C for 1 min and 72°C for 1 min, and a final extension at 72°C for 10 min. Amplified PCR products were separated by agarose gel electrophoresis, purified using QIAquick Gel (QIAGEN, UK) extraction kit and sent for direct sequencing (GATC-Biotech, Germany). The sequenced data was then verified by comparison with the genome database using BLAST from NCBI website (http://blast.ncbi.nlm.nih.gov/blast.cgi).

### Fluorescence confocal microscopy

Bladders were opened longitudinally, pinned on Sylgard plates and stretched. They were then fixed for 20–30 min in 4% neutral buffered formalin and washed in phosphate buffered saline (PBS) overnight on a shaker. Bladder sheets were cut into strips, washed and blocked with 1% horse serum in PBS+0.5% Triton X-100 (Sigma, UK) for 1 h at room temperature on a shaker. Tissues were incubated with primary antibody dilutions made up in PBS+0.5% Triton and 1% horse serum: Ano1 1∶250 (ProteinTech, Germany) or vimentin 1∶500 (Santa Cruz Biotechnology, Germany) overnight at 4°C. Negative controls omitted primary antibodies. Vascular smooth muscle present within bladder tissue was used as positive control [14]. Following several washes, tissues were incubated with 1∶750 dilutions of secondary antibodies: donkey anti-rabbit Alexa 488 for Ano1 and goat anti-mouse Alexa 594 for vimentin (Invitrogen, UK) and 2 µg/ml DAPI nuclear stain (Biotium, Germany) for 1 h at room temperature. Following several washes, whole tissues were mounted on slides with FluorSave (Calbiochem, UK). Slides were examined using a Leica SP5 confocal imaging system attached to Leica DMI 6000 inverted microscope.

### Functional Studies

Bladders were opened by a ventral incision from the urethra to the dome. Each bladder was divided into two or three longitudinal strips. Strips of denuded detrusor were prepared by gently removing mucosa (urothelium and lamina propria) under the dissecting microscope. Denuded (without mucosa) and intact (with mucosa) strips (∼2×6 mm) were mounted in 0.2 ml Perspex microbaths (Oxford University, UK) and were superfused with carboxygenated Krebs (36±1°C) at a flow rate of 2 mL/min via a peristaltic pump. Strips were equilibrated under a resting tension of 1.0–1.5 g for 60 min. Tension was monitored via isometric force transducers (Pioden Controls Ltd, UK) and a Powerlab data acquisition system running LabChart software (ADInstruments, UK). An initial priming response was elicited in all intact and denuded detrusor strips by exposure for 10 seconds to carbachol (CCh, 10 µM) (Sigma, UK) dissolved in Krebs solution. After a 10 min washout period, a 30 min control period of phasic contractile activity was recorded. Strips not showing phasic activity were discarded. Next, a single dose of a chloride channel blocker or the drug vehicle dimethyl sulfoxide (DMSO) (Sigma UK) were perfused for 30 min, with at least a 30 min washout period between the doses. The effect of 3, 10 and 30 µM NFA (Sigma, UK) and 10 and 30 µM NPPB (Sigma, UK) was investigated by measuring the amplitude (g tension per mg of tissue weight) and frequency (number of contractile events in 5 min) of phasic contractions. The threshold for a single contraction event was set at 0.05 g for intact and 0.026 g for denuded detrusor strips (modified from [15]). The amplitude and the frequency of phasic contractions were measured before (baseline) and during the last 5 min of any drug (experimental) or vehicle (control) exposure and compared using paired two-tailed Student's t-test, with p<0.05 considered statistically significant. For illustrative purposes baselines were pooled together. Percentages of inhibition of amplitude and frequency were calculated by subtracting the value at baseline from experimental and dividing it by values at baseline. I% =  ((experimental–baseline)/baseline)*100. Comparisons between intact and denuded tissues at the same drug concentration were performed using an unpaired Student's t-test, with p<0.05 considered significant. All data is presented as mean ± standard error of the mean (SEM). Statistical analysis was performed in GraphPad Prism.

## Results

### Ano1 is expressed in rat urinary bladder at mRNA level

**Figure 1:**
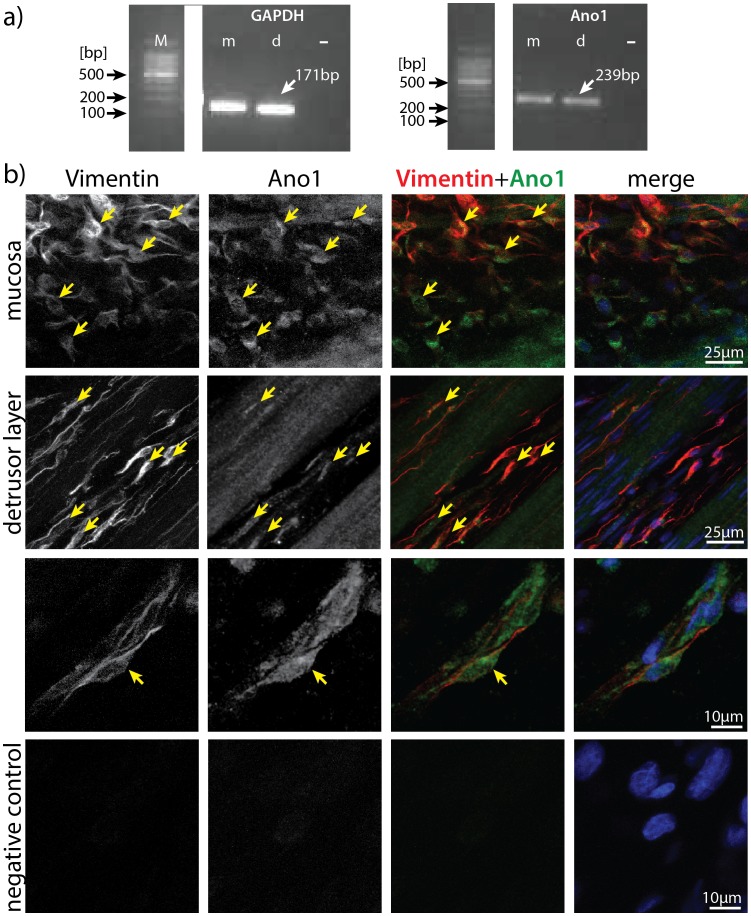
Molecular and immunohistochemical expression of Ano1 in rat urinary bladder. a) Expression of Ano1 mRNA (239 bp) was detected in detrusor (d) and mucosa (m) of rat urinary bladder; positive control was amplified with GAPDH primers (171 bp). b) Vimentin and Ano1 immunopositive cells were found in mucosa and in detrusor layer. First column shows vimentin staining; second column shows Ano1. In merged images staining shows vimentin in red, Ano1 in green and nuclei in blue. Yellow arrows show cells expressing both Ano1 and vimentin, in mucosa (first panel), detrusor layer (second panel), at higher magnification (third panel) and a negative control (fourth panel).

The presence of amplifiable cDNA product obtained from reversely transcribed mRNA extracted from mucosa and detrusor layers of rat bladders was confirmed by amplification of GAPDH and observation of a band at the expected product size (171 bp, Figure 1a). Subsequently, reverse transcription PCR demonstrated the expression of Ano1 in mucosa and detrusor layers of rat bladder (Figure 1a). Direct sequencing of the Ano1 amplicons yielded partial sequences (188–190 bp), with 99% homology with Rattus norvegicus Anoctamin-1 mRNA (accession no: NM_001107564.1) confirming the expression of Ano1 in rat bladder.

### Ano1 is co-expressed in a subpopulation of vimentin positive cells

Immunoreactivity to vimentin antibody showed a network of vimentin positive cells in the rat urinary bladder. Figure 1b shows immunostaining for vimentin on a network of connected cells between smooth muscle bundles and in mucosa of rat urinary bladder. These vimentin positive cells had elongated cell bodies and extended fine processes consistent with the morphology reported for interstitial cells of the bladder [16]. Ano1 was co-expressed on a subpopulation of vimentin immunopositive cells, both between the muscle bundles and in the mucosa (Figure 1b). Ano1 showed staining in the vascular smooth muscle as a positive control (data not shown). Negative controls without a primary antibody showed no cell specific staining (Figure 1b).

### Phasic contractions of intact vs. denuded strips from rat bladder

Rat bladder strips exhibited spontaneous phasic activity within 10 min after setting up strips in the baths. 90% (66/73) of intact strips developed spontaneous phasic contractions, while only 64% (44/68) of denuded strips showed spontaneous contractile activity. Intact strips were heavier compared to denuded detrusor strips without mucosa (Table 1). The amplitude (corrected for weight) and frequency of phasic contractions were significantly higher in intact strips vs. denuded strips (Table 1).

**Table 1:** Characterization of rat urinary bladder strips and their phasic contractions.

	weight ± SEM [mg]	amplitude ± SEM [g/mg]	Frequency ± SEM [event in 5 min]
Intact (n = 66, N = 42)	13.2 (±0.45) ***	0.04322 (±0.00527) **	27.70 (±0.86) ***
Denuded (n = 44, N = 34)	8.0 (±0.44)	0.02457 (±0.00226)	17.51 (±1.32)

Comparison of intact vs. denuded used unpaired Students t-test. Data is presented as mean±SEM.

n – strips, N – animals, SEM – standard error of the mean, ** p<.01, *** p<.001.

### Effect of NFA on phasic contractions of intact rat bladder strips

**Figure 2:**
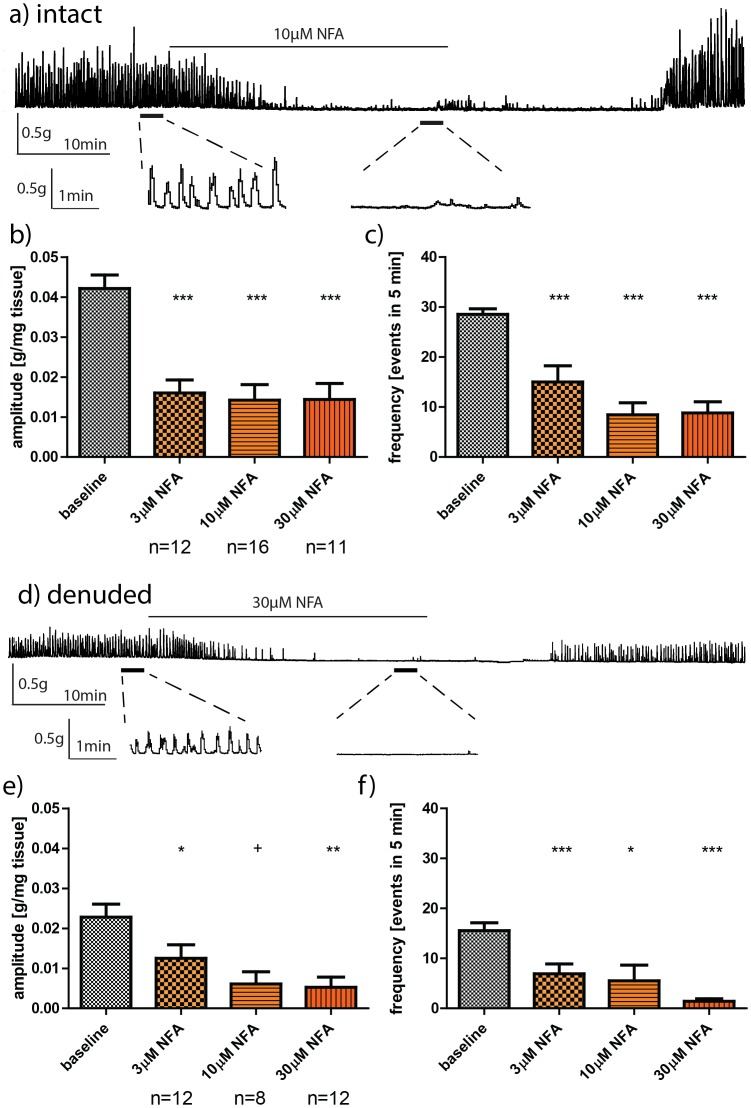
Effect of NFA on phasic activity in intact (a–c) and denuded detrusor strips (d–f). a) A representative trace of the effect of 10 µM NFA on phasic contractions of an intact strip; bars below the trace indicate expanded regions. Effect of 3 µM, 10 µM and 30 µM NFA on b) the amplitude and c) the frequency of phasic activity in intact rat bladder strips. d) A representative trace of the effect of 30 µM NFA on phasic contractions of a denuded strip; bars below the trace indicate expanded regions. Effect of 3 µM, 10 µM and 30 µM NFA on: e) the amplitude, and f) the frequency of phasic activity in denuded rat bladder strips Baselines were pooled together for illustrative purposes. Data is presented as mean±SEM. **+** p = 0.087, * p<.05, ** p<.01, *** p<.001 vs. paired baseline phasic activity

A representative trace of the effect of 10 µM NFA on the phasic activity of an intact strip is demonstrated in Figure 2a. NFA significantly (p<0.001) inhibited the amplitude and frequency of phasic activity in intact bladder strips at all concentrations tested (Figure 2b & 2c). Table 2 represents the percentage change in the amplitude and the frequency of phasic activity in intact rat bladder strips in the presence of increasing concentrations of NFA. The three tested doses of NFA (3, 10 and 30 µM) reduced the amplitude of phasic contractions in intact bladder strips by 49–70% and the frequency by 53–74% (Table 2).

**Table 2:** The percentage change in amplitude and frequency of phasic contractions in intact and denuded strips in presence of vehicle DMSO, NFA and NPPB.

drug	dose [µM]	amplitude		frequency	
		intact	denuded	intact	denuded
DMSO	0.01%	5.49±4.3%	2.93±.7.7%	2.26±6.8%	4.80±6.9%
NFA	3	−49.30±6.7%	−46.01±13.7%	−53.51±8.8%	−63.17±8.6%
	10	−66.32±10.0%	−48.66±20.6%	−71.38±8.4%	−62.28±13.9%
	30	−70.73±7.2%	−69.98±13.2%	−74.25±6.9%	−88.02±5.7%
NPPB	10	−44.00±5.5%	−33.22±7.7%	−23.39±11.3%	−44.86±7.8%
	30	−51.84±9.3%	−91.31±3.9%	−41.09±12.9%	−90.69±4.1%

Negative values indicate inhibitory effects of compounds used on phasic contractions. Data presented as mean±SEM.

### Effect of NFA on phasic contractions of denuded rat bladder strips

A representative trace of the effect of 30 µM NFA on phasic contractions of a denuded strip is demonstrated in Figure 2d. NFA (3, 30 µM) significantly (p<0.05, p<0.01) reduced the amplitude of phasic contractions in denuded strips (Figure 2e). 10 µM NFA also reduced the amplitude of phasic contractions, but this change did not reach statistical significance (p = 0.0873). NFA significantly (p<0.01–0.001) reduced the frequency of phasic contractions in denuded strips at all concentration tested (Figure 2f). The amplitude and frequency of phasic activity were reduced by 46–70% and 63–88% respectively (Table 2).

### Effect of NPPB on the phasic contractions of intact rat bladder strips

**Figure 3:**
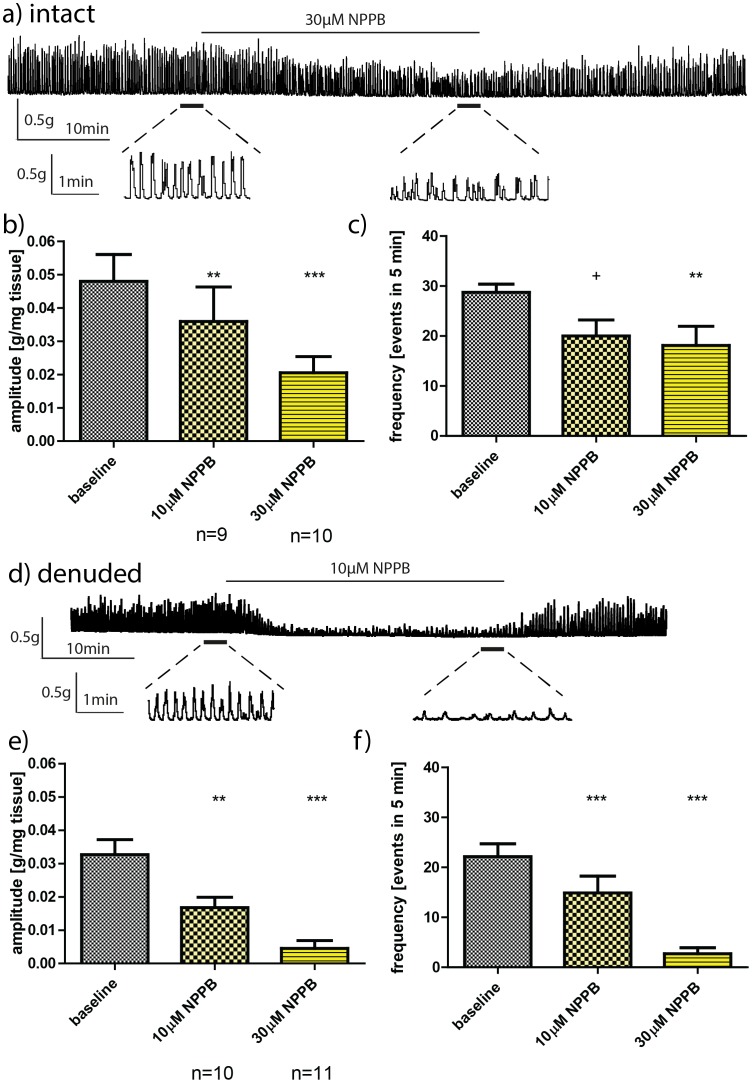
Effect of NPPB on phasic activity in intact (a–c) and denuded detrusor strips (d–f). a) A representative trace of the effect of 30 µM NPPB on phasic contractions of an intact strip; bars below the trace indicate expanded regions. Effect of 10 µM and 30 µM NPPB on b) the amplitude and c) the frequency of phasic activity in intact rat bladder strips. d) A representative trace of the effect of 10 µM NPPB on phasic contractions of a denuded strip; bars below the trace indicate expanded regions. Effect of 10 µM and 30 µM NPPB on the amplitude and f) the frequency of phasic activity in denuded rat bladder strips. Baselines were pooled together for illustrative purposes. Data is presented as mean±SEM. **+** p = 0.054 ** p<.01, *** p<.001 vs. paired baseline phasic activity.

A representative trace of the effect of 30 µM NPPB on the phasic activity of an intact strip is demonstrated in Figure 3a. NPPB (10, 30 µM) inhibited the amplitude (p<0.01, p<0.001) and the frequency (p = 0.054, p<0.01) of phasic activity in intact bladder strips (Figure 3b & 3c). Table 2 represents the percentage change in the amplitude and the frequency of phasic activity in intact rat bladder strips in the presence of increasing concentrations of NPPB. Both doses of NPPB (10 and 30 µM) reduced the amplitude and the frequency of phasic contractions in intact bladder strips, by 44–51% and 23–41% respectively (Table 2).

### Effect of NPPB on phasic contractions of denuded rat bladder strips

A representative trace of the effect of 10 µM NPPB on the phasic activity of a denuded strip is demonstrated in Figure 3d. NPPB (10, 30 µM) significantly reduced the amplitude (p<0.01, p<0.001) and the frequency of (p<0.01, 0.001) of phasic contractions in denuded strips (Figure 3e & 3f). The amplitude and frequency of phasic activity were reduced by 33–91% and 44–91% respectively (Table 2).

### Comparison of the effect of the CaCC modulators on intact vs. denuded rat bladder strips

When the percentage change in the amplitude and the frequency of phasic activity in presence of NFA and NPPB (Table 2) was compared between intact and denuded strips, there was no significant difference between these two types of strips. NFA was able to completely abolish, in a reversible manner, phasic contractions of intact and denuded strips (representative traces in Figure 2a & 2d). NPPB reduction of phasic activity was also reversible (representative traces in Figure 3a & 3d). The drug vehicle, DMSO (0.01%) had no significant effect on the amplitude or the frequency of the phasic contractions in intact (n = 27, N = 27) and denuded strips (n = 13, N = 13) (Table 2). There was no time related decline in strip phasic activity over the duration of the experimental protocol (vehicle data - Table 2).

## Discussion

Ano1 has been identified as a new, selective marker for all classes of ICCs in the stomach, small intestine and large intestine in humans and mice, allowing immunochemical identification of these cells independent of KIT [11]. In the present study expression of Ano1 was detected at the mRNA level in mucosa and detrusor layers of rat urinary bladder. Immunostaining also demonstrated the expression of Ano1 on a subpopulation of vimentin positive cells in mucosa and detrusor layers of rat bladder with morphology consistent with interstitial cells [5], [16]. The methodology was not undertaken quantitatively, but approximately 50% of cells expressing vimentin had some degree of Ano1 expression. Ano1-like immunoreactivity appeared stronger in a subgroup of the vimentin positive cells in the mucosa. The possibility of regional variation in extent of Ano1-like immunoreactivity is not precluded by the current findings.

Previous studies have also confirmed the presence of vimentin and KIT positive interstitial cells in mucosa and detrusor layers of the rat bladder wall [17], [18]. Ano1 may also provide more selective labelling of interstitial cells than KIT antibodies in the bladder, because unlike KIT, it does not stain mast cells [11]. Further characterisation of interstitial cells based on the expression profile of various cellular markers would be advantageous and would allow us to further establish their functional role. Various cell surface markers identified on bladder interstitial cells, including KIT, PDGFRα and CD34 along with specific localization of interstitial cells in the bladder wall, might suggest that distinct interstitial cells subtypes mediate various processes like signal transduction or pacemaker activity. The relationships of other interstitial cells markers like Ano1 to specific functions remains to be determined [19].

ICCs require Ano1 for generation of spontaneous transient inward current, which is a key factor driving peristalsis in the intestine [10]. Functional inhibition of CaCCs by pharmacological mediators such as NFA and NPPB impairs function of ICCs present in various segments of the intestine [9], [10]. In the urinary bladder, interstitial cells have been implicated as the modulators of its phasic activity [4]. Thus, we investigated the effects of NFA and NPPB on the phasic activity of the bladder. Both agents reduced the amplitude and frequency of phasic contractions of bladder strips. NFA was used at 3–30 µM with strong inhibitory effects observed at all tested concentrations, albeit without dose dependent effects. This concentration range has been previously used to reduce the mouse gastric antrum slow wave frequency by inhibition of Ano1 on ICCs [10].

A recent study by Lam et al. (2013) investigated the effect of NFA on phasic and agonist evoked contractions in adult rat bladder strips. 10 µM NFA increased the amplitude but not the frequency of spontaneous contractions in transversely-cut intact and denuded rat bladder strips [13]. A similar but a non-significant trend in contraction amplitude increase was detected in longitudinally cut strips. In the current study, NFA significantly reduced the amplitude and frequency of phasic contractions in longitudinally-cut bladder strips from three week old rats. Bladder phasic contractions change during neonatal development [20], changing character at 3 weeks [21] and are sensitive to stretch [22]. Therefore, the differences in ages of animals assessed and experimental protocols may explain differences seen between the two studies. In the present study, 3 week old Wistar rats were used and bladder strips were placed under 1.5 g initial tension, while Lam and colleagues used 6–7 week old Sprague-Dawley rats and continuously re-adjusted the tension of strips to 2 g.

NFA is a recognised pharmacological tool for studying CaCCs [9], [12]. However, non-specific effects on calcium and potassium channels and cyclooxygenases receptors have been suggested [14], [23], [24]. In pulmonary vein smooth muscle tissue, NFA off-target effects are not significant [14]. Nonetheless, the current study additionally employed NPPB to evaluate the hypothesis that CaCCs play a role in mediating phasic activity of the rat urinary bladder. NPPB inhibited the phasic contractions of rat bladder strips at all concentrations tested, consistent with the effects of NFA. 30 µM NPPB inhibited the amplitude and frequency of phasic activity by up to 90%. In other tissues, including gut ICCs and airway smooth muscle, NPPB blocks chloride currents attributed to CaCCs [9], [25] at a range 50–100 µM. More targeted pharmacological agents are needed to determine the role of Ano1 in modulation of bladder contractions. To date, Ano1 inhibitor T16Ainh-A01 has been found as a specific Ano1 blocker [26] and used in other tissues [14]. Commercially available T16Ainh-A01 (Tocris, UK) formed precipitate in the buffer during drug exposure in our experimental setup, making it impossible to conduct controlled experiments due to poor tissue penetration by the drug, lack of access to target cells or problems with dissolving of the compound.

A previous study on interstitial cells of the mucosa in the guinea-pig bladder demonstrated CaCC currents were attenuated by CaCC blocking agent 4,4'-Diisothiocyano-2,2'-stilbenedisulfonic acid (DIDS) [8]. In the present study, there were no significant mucosa dependent differences in bladder responses to CaCC modulators, suggesting that the CaCC expressed on interstitial cells of the mucosa and the detrusor layers may be equally important in mediating the phasic activity of rat bladder strips. However, other CaCCs have been found at the mRNA level in the detrusor layer of various species [27]–[29]. In the urethra, Ano1 was found to be expressed on smooth muscle cells [30] but not on urethral interstitial cells and NFA was able to inhibit the urethral smooth muscle contractile activity in mouse, rat and sheep [30]. Therefore, we cannot rule out the expression of other CaCCs on rat bladder smooth muscle cells and the possible effect of NPPB and NFA on these channels. Further studies are needed to distinguish between the effects of CaCC modulators on bladder interstitial vs. smooth muscle cells. The exact role of Ano1 channel on the excitability of interstitial cells at the channel level also remains to be elucidated.

## Conclusions

We demonstrated that Ano1 is expressed at the mRNA level in the young rat urinary bladder. Ano1 was present in a subpopulation of vimentin positive interstitial cells in mucosa and detrusor layers.

Pharmacological inhibition of Ano1 by CaCC blockers reduced the amplitude and frequency of phasic activity of intact and denuded bladder tissue strips, providing a new pharmacological tool for modulating phasic activity.
